# Screening for Systemic Light-Chain Amyloidosis in Patients Over 60 with λ Monoclonal Gammopathies

**DOI:** 10.3390/jcm14124146

**Published:** 2025-06-11

**Authors:** Ping Zhou, Mahesh M. Mansukhani, Raymond Yeh, Jiesheng Lu, Hongai Xia, Lahari Koganti, Jiuhong Pang, Denis Toskic, Stephanie Scalia, Xun Ma, Lisa X. Lee, Sandy W. Wong, Alfred Chung, Sascha A. Tuchman, Terry Fogaren, Nancy Coady Lyons, Cindy Varga, Suzanne Lentzsch, Raymond L. Comenzo

**Affiliations:** 1 The Tufts Medicine Myeloma and Amyloid Program, Tufts Medical Center, 800 Washington Street, P.O. Box 826, Boston, MA 02111, USA; ping.zhou@tuftsmedicine.org (P.Z.); denis.toskic@tuftsmedicine.org (D.T.); stephanie.scalia@tuftsmedicine.org (S.S.); xun.ma@tuftsmedicine.org (X.M.); teresa.fogaren@tuftsmedicine.org (T.F.); nancy.lyons@tuftsmedicine.org (N.C.L.); cindy.varga@atriumhealth.org (C.V.); 2 Columbia University Laboratory of Personalized Genomic Medicine, Department of Pathology & Cell Biology, Columbia University Irving Medical Center (CUIMC), New York, NY 10032, USA; mm322@cumc.columbia.edu (M.M.M.); ry2261@cumc.columbia.edu (R.Y.); jl937@cumc.columbia.edu (J.L.); hx2199@cumc.columbia.edu (H.X.); lk2776@cumc.columbia.edu (L.K.); jp3117@cumc.columbia.edu (J.P.); 3 Chao Family Comprehensive Cancer Center, University of California, Orange, Irvine, CA 92617, USA; lisaxl@hs.uci.edu; 4 Helen Diller Family Comprehensive Cancer Center, University of California, San Francisco, CA 94143, USA; sandy.wong.md@gmail.com (S.W.W.); alfred.chung@ucsf.edu (A.C.); 5 Multiple Myeloma and Amyloidosis Program, Division of Hematology, Department of Medicine, University of North Carolina, Chapel Hill, NC 27599, USA; sascha_tuchman@med.unc.edu; 6 Division of Hematology-Oncology, Department of Medicine, Tufts Medical Center, Boston, MA 02111, USA; 7 Multiple Myeloma and Amyloidosis Service, CUIMC, New York, NY 10032, USA; sl3440@cumc.columbia.edu

**Keywords:** smoldering myeloma, AL amyloidosis, screening

## Abstract

**Background/Objectives:** To reduce the early mortality of light-chain amyloidosis (AL), earlier diagnosis is needed. To pursue this goal, we conducted a multicenter study screening for AL λ-type (NCT04615572) in subjects > 60 years of age with λ smoldering myeloma (SMM) or monoclonal gammopathy of undetermined significance (MGUS), a light-chain differential (dFLC, λ minus κ) > 23 mg/L, and no prior amyloid diagnosis. **Methods**: Variables included AL-related IGVL gene usage and clonal plasma cell cytogenetic abnormalities, such as t(11;14) or gain 1q, which are present in 75% of AL cases. Here, 9 out of 33 λ IGVL genes, accounting for 90% of AL λ cases, were considered to be AL-related. Bone marrow was obtained, plasma cell cytogenetics and next generation sequencing for IGVL genes were performed, and subjects with AL-related IGVL genes were screened for AL using tissue studies. **Results**: From 2021 to 2023, we enrolled 30 subjects (19 M/11 F) with a median age of 68.5 years old (IQR 64.3–73), 17 SMM and 13 MGUS, with a median of 6% marrow plasma cells (range, 3.5–40). Here, 11 SMM and 4 MGUS cases had t(11;14) or gain 1q; 10/17 SMM and 12/13 MGUS had AL-related genes, and AL was ultimately confirmed by tissue biopsy in 3 with SMM. SMM, AL-related IGVL genes, and t(11;14) or gain 1q were found in 6 SMM subjects, including the 3 with AL (3/6 vs. 0/16; *p* < 0.05, Fisher’s exact, two-tailed). **Conclusions**: These results justify a larger study screening for AL in SMM to develop a likelihood algorithm for AL using dFLC, IGVL gene usage, and the presence of t(11;14) or gain 1q.

## 1. Introduction

Delays in the diagnosis of AL occur even in patients with SMM, a known risk factor for the disease. Early diagnosis is critical given the frequency of early cardiac death and the availability of effective therapies [1,2,3]. SMM patients have an M-protein of >3 g/dL or >10% clonal plasma cells in the marrow, without myeloma-related end-organ damage. They are monitored expectantly for progression to MM requiring treatment. Progression to AL is not a reliable focus of expectant observation, and AL diagnosis is often too late [4,5,6]. In the USA, in 2023, the American Cancer Society estimated there would be 35,730 new cases of MM. Of them, 15% would have SMM, 2% of whom would progress to AL; in time, 12% to 15% of MM patients will develop AL. Nevertheless, patients with SMM continue to have delays in the diagnosis of AL. Experienced hematologists looking after patients with SMM who develop dyspnea, lower extremity edema, postural symptoms, and weight loss often refer them to other specialists who routinely miss the diagnosis of AL [7,8,9].

This is particularly troubling because the presence of a monoclonal gammopathy can be a precursor phase to AL, and without early recognition, the organ disease can progress rapidly [9]. In a study employing pre-diagnostic specimens from members of the US Armed Forces (DoD) subsequently diagnosed with systemic AL amyloidosis, investigators showed that for a decade prior to diagnosis, 80% had monoclonal serum free light-chain (FLC) abnormalities [10]. This precursor phase is characterized by the presence of a monoclonal gammopathy with differential free light-chain values (involved—uninvolved = dFLC) > 23 mg/L [10,11,12]. In the DoD series, upon diagnosis of AL, 55% of patients had cardiac involvement, a harbinger of mortality [10]. Of such cases, 20% of patients die within 6 months despite treatment, an outcome that has not improved in 40 years [13]. Moreover, in a multivariate analysis of risk factors for cardiac-related mortality in newly diagnosed AL patients, older age significantly predicted risk of death [14]. Of note, the median age at diagnosis in patients with AL is 63 years [15].

In a pilot internet-based study screening for AL in patients with λ monoclonal gammopathies, we identified undiagnosed AL in 2 of 19 subjects with SMM using dFLC and AL-related IGVL as screening variables [12]. In this multicenter study, we used the presence of AL-related IGVL genes to screen for the presence of AL in patients > 60 years of age with λ SMM and λ MGUS with a dFLC > 23 mg/L. We also gathered data on the cytogenetic abnormalities t(11;14) and gain 1q frequently found in AL. We chose to focus this study on AL λ-type because λ-type is 3× more common than κ-type.

## 2. Patients and Methods

### 2.1. Patients

To be eligible for this multi-center study (NCT04615572), subjects had to be 60 years of age or older and had to have λ isotype SMM or MGUS with the difference between λ and κ FLC > 23 mg/L, a κ:λ FLC ratio below 0.26 and no prior biopsies showing amyloid. In patients with eGFR < 50, the κ:λ FLC ratio criterion was waived because clearance of κ FLC decreases as renal function worsens, increasing the κ FLC levels in the blood and increasing the ratio [16]. The criteria for MGUS were a monoclonal protein (M-protein) and < 10% marrow plasma cells and for SMM an M-protein of >3 g/dL or >10% clonal marrow plasma cells and, in both cases, no myeloma-related end-organ damage. Cytogenetic studies were performed locally at the collaborating centers, enabling the identification of t(11;14) and gain 1q with the marrow cells obtained upon enrollment.

The aims of the study were to determine the likelihood of having AL in patients with λ MGUS or λ SMM by screening for dFLC > 23 mg/L and AL-related IGVL identification leading to tissue biopsy. We also obtained cytogenetic results for the presence of t(11;14) and gain 1q. In addition, we sought to determine if marrow mononuclear cells were adequate for λ IGVL germline gene identification by next generation sequencing compared to immunomagnetically selected plasma cells (CD138-selection). Standard epidemiologic and clinical laboratory and bone marrow data were also obtained.

### 2.2. IGVL Gene Identification

λ IGVL identification was performed on the Illumina platform in the Columbia University Irving Medical Center (CUIMC) Personalized Genomic Medicine Laboratory using 9 variable region and 2 constant region primers. The resulting PCR products were sheared by ultrasonication and, following end-repair and adapter ligation, sequenced on a MISeq (Illumina, San Diego, CA, USA) using a 300-cycle paired-end flow cell. After demultiplexing, Fastq files were converted to FASTA sequences and mapped for IGVL segments using IMGT (the international ImMunoGeneTics information system)-Quest to obtain clonal reads. We also performed RT-PCR for λ IGVL identification with CD138-selected cell cDNA, as previously described and used IMGT search functions for IGVL gene identification [17]. In 2020, the determination that a set of 9 of 33 λ IGVL genes were AL-related, covering over 90% of AL cases, was based on the data in AL-Base at that time (AL-Base is a platform analysis tool for the study of amyloid-forming immunoglobulin light-chain sequences [18]).

### 2.3. Biostatistics

Statistical analyses were performed with Graphpad Prism V5 (GraphPad, San Diego, CA, USA).

## 3. Results

### 3.1. Patients

From mid-2021 to early 2023, we enrolled 30 subjects. We initially aimed for 50 subjects, but the pandemic and funding limited presented limitations. Instead, 19 M/11 F with a median age of 68.5 years (58–92) completed the assessment of marrow specimens (Table 1).

### 3.2. Marrow Findings

Seventeen subjects with SMM had a median of 10.5% clonal plasma cells (range, 10–40%) and eleven had t(11;14) or gain 1q. Thirteen subjects with MGUS had a median of 5.5% clonal plasma cells (range, 3.5–7%) and four had t(11;14) or gain 1q. λ IGVL genes were identified with BM MNC cDNA in 29 of 30 subjects (MGUS =13/13, SMM = 16/17); RT-PCR identified the one remaining SMM IGVL gene.

**Figure 1:**
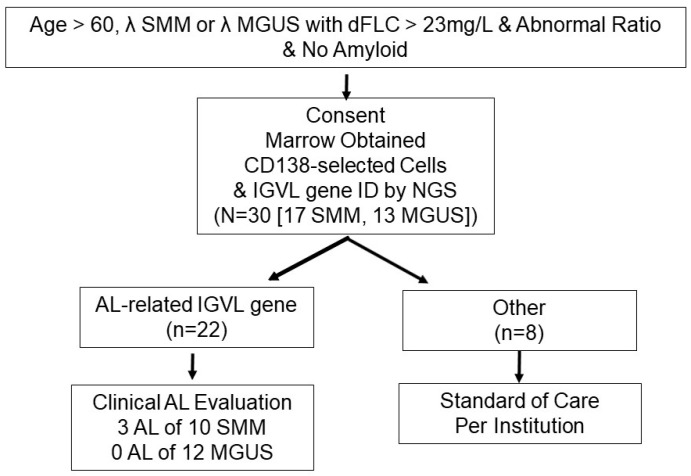
Screening Schema.

Of the 17 SMM patients, 10 had AL-related genes, as did 12 MGUS patients, all 22 of whom were screened for AL with tissue biopsies (Table 2). The other 8 subjects of the 30 were asymptomatic and had marrows that were negative for amyloid, but they were not screened with other tissue biopsies. AL was found in 3 SMM patients and no others, as summarized in Figure 1.

AL-related IGVL genes were found in 22 subjects. Analysis using a two-tailed Fisher’s exact test of SMM compared to MGUS shows a trend toward significance (3/10 vs. 0/12; *p* = 0.08). When using AL-related IGVL genes, SMM, and t(11;14) or gain 1q—found in 6 subjects, including 3 ultimately discovered to have AL—the result achieved statistical significance (3/6 vs. 0/16; *p* < 0.05, Fisher’s exact, two-tailed). In contrast, examining SMM with t(11;14) or gain 1q alone did not reach significance (3/11 vs. 0/6; *p* > 0.05). Although these statistical findings are modest, it is important to note that the exploratory nature of this study helped establish testable hypotheses and variables for developing a likelihood algorithm to detect AL in SMM subjects.

### 3.3. Subjects Found to Have AL

The three subjects with AL had LV2-23 in one case and LV3-1 in the other two. One was a 70-year-old man from New York with a recent diagnosis (within 1 month) of SMM with t(11;14), who presented with penile ecchymoses and was found to have AL λ-type by fat pad aspirate and mass spectrometry. Upon diagnosis, he had cardiac stage II involvement with an N-terminal prohormone brain natriuretic peptide (NT-proBNP) level of 1916 pg/mL but was asymptomatic. He received Daratumumab-based therapy and achieved a complete hematologic response with a decrease in NT-proBNP to 1066 pg/mL. The second patient, a 60-year-old man from Tennessee, had peripheral neuropathy and a 127-month history of SMM with t(11;14). Amyloid was detected in his abdominal fat, but the sample was insufficient for typing. He was treated with venetoclax-based therapy and achieved a complete response within a year, along with subjective improvement in his peripheral sensory neuropathy. The third subject was a 71-year-old woman from California with a 17-month history of SMM, t(11;14), and marrow findings consistent with λ-type amyloid, prompting further evaluation. Her NT-proBNP was 809 pg/mL, and cardiac MRI showed findings consistent with amyloid cardiomyopathy. She received daratumumab-based therapy, with venetoclax added after a sub-optimal response, followed by a stem-cell transplant with 140 mg/M^2^ of melphalan. She subsequently achieved a cardiac response and a reduction in FLC from 888 to 84 mg/L. No MGUS patients were found to have AL. One SMM patient (with LV1-44) progressed to MM requiring therapy.

### 3.4. Marrow Mononuclear Cells (MNC)

From 2 × 10^6^ marrow MNC, we obtained 1108 ng/μL cDNA (540–2299) and from CD138-selected cells we obtained 1184 ng/μL (484–1986). We sent 60 μL of each sample in blinded fashion to CUIMC. The privacy and confidentiality standards were stringent and highlighted on the informed consent document. The IGVL genes identified in both samples matched for all patients, although the number of CDR3 reads were higher using CD138-selected cell cDNA with a mean (±SD) of 5.3 ± 2.9 × 10^4^ compared to 3.9 ± 2.6 × 10^4^ using MNC cDNA (*p* << 0.01, paired *t* test, two-tailed). In the 17 SMM cases, NGS was non-productive in one case with both MNC and CD138-selected cell cDNA; RT-PCR identified the clonal gene. A single IGVL gene was identified in MNC cDNA in 15, and both LV1-44 and LV1-47 were amplified in 1 case with reads of 21,683 for the former and 67,886 for the latter and similar findings with CD138-selected cell cDNA. In 10 of 13 MGUS cases, one IGVL gene was identified in MNC cDNA, while in two cases, 2 genes were amplified with different CDR3 regions, and in another case, 3 were amplified (ranked by Reads LV1-44 > LV3-19 > LV10-54). The implications of monoisotypic multiclonal gammopathies are not known. In conclusion, CD138-selection was not needed to identify the clonal genes in these cases; marrow mononuclear cells were sufficient.

## 4. Discussion

Earlier detection of AL remains an unmet need [10]. Advances in order to meet this need will involve determining the likelihood or risk of AL in patients with monoclonal gammopathies, such as SMM. In SMM, 23% of patients have t(11;14) and 30% had gain 1q [6,19,20,21]. In a series of 133 AL patients whose marrow plasma cells were evaluated for clonal cytogenetic abnormalities, 83 (62%) had t(11;14) and 35 of 130 (27%) had gain 1q [15]. In another series of 140 AL patients, 59% had t(11;14) and 20% had gain 1q [22]. On average, 60% of AL cases are t(11;14) and 29% are gain 1q. The t(11;14) translocation, deleting the heavy chain locus, likely accounts for the high frequency of FLC-producing clones in AL [18,23].

In addition to at least 75% of AL patients having either t(11;14) or gain 1q, there is a 3:1 λ isotype predominance and relatively restricted λ IGVL gene usage [12,24]. Among the 33 λ IGVL germline genes on chr 22q11.2, nine accounted for 93% of the AL λ-type IGVL sequences in the Boston University database in 2020 (579 of 620), and 87% of the λ IGVL sequences in the Mayo Clinic report based on biopsy-derived protein sequences from patients (371 of 428) [12,24]. At the start of this study, we designated these 9 genes (LV6-57, LV2-14, LV1-44, LV3-1, LV1-51, LV3-21, LV3-19, LV2-23, LV1-40) as being related to AL λ-type. In a prior internet-based screening study, we identified two cases of AL requiring therapy in 21 patients with λ monoclonal gammopathies who were screened based on IGVL gene identification (both were SMM and had LV2-14) [12]. Recently, AL-Base has been updated to include over 2000 monoclonal light-chain sequences from myeloma, AL, and other plasma cell dyscrasias. Sixteen germline sequences (12 λ and 4κ) were significantly associated with AL compared to myeloma and other sequences, including LV2-23 and LV3-1, the IGVL genes found in the three AL patients identified in this study [25].

In this multicenter study, we used age, the FLC criterion of a dFLC > 23 mg/L, and the presence of AL-related IGVL genes to evaluate the screening results for the presence of AL in patients with λ SMM and MGUS. Twenty-two of thirty subjects who met the age and FLC criteria had AL-related IGVL genes and were screened for AL with tissue biopsies. This raises a question regarding the utility of AL-related IGVL genes as a selection variable; however, as the contingency table analyses above suggest, IGVL germline gene use in AL may be a key variable to include when attempting to create a likelihood algorithm for AL in SMM patients. Of note, a recent re-evaluation of AL-Base that included a significantly increased number of IGVL sequences from myeloma patients concluded that 8 of the 9 genes we determined were AL-related were significantly over-represented in AL compared to multiple myeloma [25]. Moreover, our study confirms that marrow mononuclear cells shipped overnight to a central facility were adequate for the molecular identification of IGVL genes by next generation sequencing without the need for a CD138-selection step.

The additional variable NT-proBNP, not collected in this study, may provide further predictive power to a likelihood algorithm [26]. A larger study, in addition to adding NT-proBNP, could also focus on subjects with SMM of both isotypes, κ and λ, with FLC gene identification for both isotypes; exclude MGUS; lower the age for eligibility; and employ tissue testing for AL upon enrollment of all subjects with SMM and dFLC > 23 mg/L. Screening for AL κ-type in subjects with SMM increases the complexity of NGS. There are more κ IGVL genes (about 40) than λ, and the AL κ-type repertoire is less restricted. But, primers and methods are available [27]. In SMM, 70% of patients have κ-type serum M-proteins, with 53% employing KV1 family genes, although the frequency is only 50% for those with Bence−Jones proteinuria containing light chains [28,29]. In contrast, 67% of AL κ-type employ KV1 genes. In systemic AL cases reported by investigators at Mayo Clinic, there were 185 κ-type, 124 (67%) of which were KV1; KV3-15 and KV4-01 accounted for 33 of the remaining cases. In that data set, KV1-16, KV1-33, and KV1-39 accounted for 69 (56%) of KV1 cases [24]. In AL-Base, KV1-16 and KV1-33 accounted for 10% and 40%, respectively, of KV1 AL genes. In our original assessment of IGVL genes and organ tropism, all 12 κ cases were KV1 family members [30]. For a large study, we could reasonably consider KV1 genes and KV3-15 and KV4-01 as AL-related since they accounted for 85% of AL κ-type cases in the Mayo data set [24]. Another consideration involves the use of the dFLC of 23 mg/L; 29 of the 30 cases we reported had abnormal ratios, but of course these cases were all λ. Given the changes in the ratio that occur with renal insufficiency, which affect κ light-chain measurements, it is likely more appropriate to use the dFLC based on the DoD data [10,16].

In addition to the use of biomarkers for screening, the evaluation of cardiac involvement in patients diagnosed with AL—such as the case of the 71-year-old woman described above and the 59-year-old woman with SMM for 9 years who was found to have cardiac involvement with AL in our first screening study—requires both advanced echocardiography and often cardiac magnetic resonance imaging [31,32,33].

Major limitations of our study are that the sample size is small and that not all subjects were screened for AL. The latter limitation clearly may have introduced bias and underestimated the presence of AL in this small sample. Nevertheless, in this exploratory study, the cases are drawn from multiple centers across the country and the identification of three cases of AL in patients with SMM, t(11;14) and predefined AL-related IGVL genes supports the pursuit of a larger study (Table 3). All three patients with AL in this study were treated and achieved hematologic responses and clinical improvement. Notably, all three AL patients had SMM, as did the two patients in our prior online study, which guided us in the design of a larger study considering SMM instead of MGUS subjects (R01-CA279808) [12].

Early diagnosis of AL may require the creation of novel approaches such as electron microscopy for light-chain ‘seeds’ in surrogate biopsies or the imaging of amyloid with agents such as 18F-florbetapir or designer peptides [34,35]. The latter may enable imaging similar to radioactive pyrophosphate scans used for diagnosing hereditary and wild-type transthyretin cardiac amyloidosis without need for a biopsy [36]. A screening model such as the one proposed here may also be useful as an initial step before expensive imaging studies. A similar model for Alzheimer’s disease (AD) is the PrecivityAD test for beta-amyloid and for Apo E isoform-specific prototyping, which has been approved by the FDA as a screening test to assist in identifying relative risk of AD and in selecting patients for expensive 18F-florbetapir scans [37]. Finally, our results support the relevance of using the following parameters in developing a likelihood algorithm for AL or risk of AL in SMM patients: a dFLC of > 23 mg/L (based on the DoD cases), the presence of t(11;14) or gain 1q, NT-pro BNP > 332pg/mL and AL-related IGVL genes.

## Figures and Tables

**Table 1:** Baseline characteristics of subjects in NCT04615572 (*n* = 30).

Age	Characteristic
Median (range) years	69 (58–92)
Distribution—no. (%)	
<65 y	7 (23)
≥65 y	23 (77)
Sex, no. (%)	
Male	19 (63)
Female	11 (37)
Median months from SMM diagnosis to enrollment	13 (1–132)
Median serum creatinine (normal 0.55–1.3 mg/dL)	1.0 (0.60–3.08)
Median alkaline phosphatase (normal 30–130 U/L)	81 (37–180)
MGUS/SMM (*n*)	13/17
MGUS	
Median iFLC (mg/L)	326 (42–1328)
Median FLC ratio	0.11 (0.02–0.30)
t(11;14) (number)	1
gain 1q21 (number)	3
SMM	
Median iFLC (mg/L)	201 (83–1040)
Median FLC ratio	0.06 (0.01–0.26)
t(11;14) (number)	6
gain 1q21 (number)	6 *

* One subject had both t(11;14) and gain 1q. Total patients was 11.

**Table 2:** NGS IGVL gene identifications in λ SMM subjects.

SMM IGVL	NCT04615572 (*n* = 17 SMM)	GenBank #
LV1-44	2	OQ912884 OQ912876
LV1-47	2	OQ884472 OR506910
LV2-8	3	OQ912883 OQ912886 OQ912887
LV2-11	1	OQ912882
LV2-14	1	OQ912877
LV2-23	2 **(1 AL)**	OR506909 **OQ912881**
LV3-1	3 **(2 AL)**	OQ819165 **OQ912879** **OQ912885**
LV3-12	1	OQ912875
LV3-21	2	OQ912880 OQ912878

**Table 3:** Characteristics of the SMM subjects who had AL-related IGVL genes.

Age/Sex	SMM	dFLC>23mg/L	AL-Related Gene	t(11;14) or Gain 1q	AL	AL Cardiac *
70M	√	√	√	√	√	√
60M	√	√	√	√	√	
71F	√	√	√	√	√	√
73M	√	√	√	√		
67M	√	√	√	√		
65M	√	√	√	√		
92M	√	√				
66M	√	√				
67M	√	√				

* NT-proBNP > 332pg/mL. √ = present.

## Data Availability

The data that support the findings of this study are openly available in the Harvard Dataverse at https://doi.org/10.7910/DVN/RI83WG (accessed on 3 June 2025). This study was posted on ClinicalTrials.gov (NCT04615572).
